# *FEN1* gene variants confer reduced risk of breast cancer in chinese women: A case-control study

**DOI:** 10.18632/oncotarget.12948

**Published:** 2016-10-27

**Authors:** Shuai Lin, Meng Wang, Xinghan Liu, Ye Lu, Zhuoqing Gong, Yan Guo, Pengtao Yang, Tian Tian, Cong Dai, Yi Zheng, Peng Xu, Shanli Li, Yuyao Zhu, Zhijun Dai

**Affiliations:** ^1^ Department of Oncology, Second Affiliated Hospital of Xi'an Jiaotong University, Xi'an 710004, China; ^2^ Department of Student Affairs, Second Affiliated Hospital of Xi'an Jiaotong University, Xi'an 710004, China; ^3^ Department of Health Science Center, Xi'an Jiaotong University, Xi'an 710061, China; ^4^ Key Laboratory of Biomedical Information Engineering of Ministry of Education, School of Life Science and Technology, Xi'an Jiaotong University, Xi'an 710049, China

**Keywords:** flap endonuclease 1, breast cancer, risk, case-control study

## Abstract

This study aimed to assess the associations of two common Flap endonuclease 1 (*FEN1*) polymorphisms (rs4246215 and rs174538) with breast cancer risk in northwest Chinese women. We conducted a case-control study with 560 breast cancer patients and 583 age-matched healthy controls from Northwest China. Odds ratios (ORs) and 95% confidence intervals (95% CIs) were used to estimate the associations. We found a significantly reduced risk of breast cancer associated with T allele of rs4246215 (allele model: OR 0.81, 95% CI 0.68–0.96; homozygote model: OR = 0.59, 95% CI = 0.40–0.87; recessive model: OR = 0.61, 95% CI = 0.42–0.89), especially in postmenopausal women (OR = 0.58, 95% CI = 0.35–0.97). Furthermore, the polymorphism showed a decreased association with larger tumor size (heterozygote model: OR = 0.63, 95% CI = 0.44–0.92; dominant model: OR = 0.63, 95% CI = 0.44–0.90). For rs174538, we did not find any difference in all genetic models. However, rs174538 was associated with lymph node metastasis (heterozygote model: OR = 0.57, 95% CI = 0.39–0.81; dominant model: OR = 0.61, 95% CI = 0.43–0.86) and estrogen receptor status (heterozygote model: OR = 1.50, 95% CI = 1.05–2.15; dominant model: OR = 1.42, 95% CI = 1.01–1.98). Haplotype analysis showed that T_rs4246215_G_rs174538_ haplotype was a protective factor of breast cancer (OR = 0.34, 95% CI = 0.14–0.81). Our results suggest that *FEN1* polymorphisms may reduce the risk of breast cancer in Chinese women.

## INTRODUCTION

Breast cancer (BC) is the most frequently diagnosed cancer and the leading cause of cancer death among females worldwide [1]. It was estimated that there were 268,600 new cases and 69,500 deaths in Chinese women in 2015 [2]. And, 1,685,210 new cancer cases and 595,690 cancer deaths are projected to occur in the United States in 2016 [1]. Many factors, such as family history, living habits, and emotional expression, are identified as potential risk factors for the development of BC [3, 4]. Recent studies also indicated that the risk of BC may be affected by genetic alterations, including genetic polymorphisms [5].

Genome stability is vital in the transmission of genetic information, and DNA molecule is vulnerable to factors that can induce DNA damage or block replication [6]. Flap structure-specific endonuclease 1 (*FEN1*) has been well characterized as a key factor in ensuring genomic stability and protecting tissues from tumorigenesis [7]. Human *FEN1*, located on chromosome 11q12, contains two exons and one intron. *FEN1* is essential for the long-patch pathway, which conducts a repair tract of at least two nucleotides. It plays an important role in the following processes: DNA repair by removing the 5′-flaps generated by Pol δ/ε [8], primer removal during lagging-strand DNA synthesis and Okazaki fragment processing [9], and the promotion of apoptotic DNA fragmentation after apoptotic stimuli. It has been reported that *FEN1* expression is related to the development and progression of various cancers [10]. Previous studies demonstrated that *FEN1* overexpression is common in breast [11, 12], prostate [11], lung, and brain tumors [13], predicting *FEN1* might be a marker of tumor progression for many types of tumors [14].

Previous studies have suggested that single nucleotide polymorphisms (SNPs) in *FEN1* may confer susceptibility to cancer. Two SNPs, 4150G>T (rs4246215, in the gene 3′-untranslated region) and −69G>A (rs174538, in the gene promoter region), were identified after thoroughly resequencing the *FEN1* locus in 30 Han Chinese healthy volunteers [15]. There have also been several studies that have investigated the associations between these two SNPs and cancer risk [15–18]. And, up till now, there was only one study performed by Lv's et al. referring to BC [18]. It was lack of repeated research to verifying the relationship between these two common SNPs of *FEN1* and breast cancer. Therefore, we conducted a case-control study to investigate the associations of the *FEN1* rs4246215 and rs174538 polymorphisms with BC risk in the Northwest Chinese population.

## RESULTS

### Characteristics of the study participants

Basic clinical characteristics of BC patients and the demographic characteristic of both the patients and healthy controls are presented in Table 1. There is no statistically significant difference between the two groups in age (*P* = 0.612) or the distribution of menopausal status (*P* = 0.716). However, the BMI was significantly different between BC patients and healthy controls (*P* = 0.038).

**Table 1:** Distributions of select variables in BC patients and cancer-free controls

Characteristics		Cases	Control	*P* value^*^
**Number**		560	583	
**Age (mean ± SD)**		49.09±11.02	48.80±8.28	0.612
**Menopausal status**				
**Premenopausal**		264	281	
**Postmenopausal**		296	302	0.716
**Body mass index (kg/m^2^)**				
**(mean ± SD)**		22.52±2.84	22.95±3.21	0.038
**Tumor size**	<2 cm	188		
	≥2 cm	372		
**LN metastasis**	Negative	236		
	Positive	324		
**ER**	Negative	247		
	Positive	313		
**PR**	Negative	255		
	Positive	305		
**Her-2**	Negative	389		
	Positive	171		
**Ki67**	< 14%	195		
	≥ 14%	365		

* T-test or two-sided χ^2^-test.

LN: Axillary lymph node, ER: Estrogen receptor, PR: Progesterone receptor, HER-2: human epidermal growth factor receptor 2.

### Association between *FEN1* polymorphisms and BC risk

The genotype and allele frequencies of the *FEN1* rs4246215 and rs174538 polymorphisms are shown in Table 2. The genotype frequencies of the two SNPs in controls both conformed to HWE (*P* = 0.253, 0.922 for rs4246215 and rs174538, respectively). Compared with the GG genotype, the TT genotype frequency of the rs4246215 polymorphism among patients was significantly different from that of controls (OR = 0.59, 95% CI = 0.40–0.87, *P* = 0.007, corrected *p* = 0.014). The difference in the frequency distributions of T and G alleles among patients and controls was also significant (OR = 0.81, 95% CI = 0.68–0.96, *P* = 0.02, corrected *p* = 0.04). Compared to individuals with rs4246215 GG/GT genotypes, individual with TT genotypes had significantly decreased BC risk (OR = 0.61, 95% CI = 0.42–0.89, corrected *p* = 0.018). These results suggested that the *FEN1* rs4246215 polymorphism had a protective effect on BC. However, we did not observe any significant associations between the *FEN1* rs174538 polymorphism and BC risk in any genetic model (shown in Table 2).

**Table 2:** Genotype and allele frequencies of *FEN1* polymorphisms among the cases and controls and the associations with BC risk

Model	Genotype	Case(560)	Control(583)	OR (95% CI)^†^	*P*-value^*^
**rs4246215**					
**Codominant**	G/G	260 (46.4%)	245 (42.0%)	1.00 (reference)	**0.026**
	G/T	249 (44.5%)	256 (43.9%)	0.92 (0.72-1.17)	0.49
	T/T	51 (9.1%)	82 (14.1%)	0.59 (0.40-0.87)	**0.007**
**Dominant**	GG	260 (46.4%)	265 (43.9%)	1.00 (reference)	
	G/T-T/T	300 (53.6%)	338 (56.1%)	0.84 (0.66-1.06)	0.13
**Recessive**	G/G-C/T	509 (90.9%)	501 (85.9%)	1.00 (reference)	
	T/T	51 (9.1%)	82 (14.1%)	0.61 (0.42-0.89)	**0.009**
**Overdominant**	G/G-C/T	311 (55.5%)	327 (56.1%)	1.00 (reference)	
	G/T	249 (44.5%)	256 (43.9%)	1.02 (0.81-1.29)	0.85
**Allele**	G	769 (68.7%)	746 (64.0%)	1.00 (reference)	
	T	351 (31.3%)	420 (36.0%)	0.81 (0.68-0.96)	**0.018**
**rs174538**					
**Codominant**	G/G	243 (43.4%)	233 (40.0%)	1.00 (reference)	0.32
	G/A	256 (45.7%)	272 (46.6%)	0.90 (0.70-1.16)	0.42
	A/A	61 (10.9%)	78 (13.4%)	0.75 (0.51-1.10)	0.14
**Dominant**	G/G	243 (43.4%)	233 (40.0%)	1.00 (reference)	
	G/A-A/A	317 (56.6%)	350 (60.0%)	0.87 (0.69-1.10)	0.24
**Recessive**	G/G-G/AG	499 (89.1%)	505 (86.6%)	1.00 (reference)	
	A/A	61 (10.9%)	78 (13.4%)	0.79 (0.55-1.13)	0.20
**Overdominant**	G/G-G/AG	294 (53.5%)	311 (53.3%)	1.00 (reference)	
	A/A	256 (46.5%)	272 (46.7%)	0.96 (0.76-1.22)	0.75
**Allele**	C	742 (66.3%)	738 (63.3%)	1.00 (reference)	
	T	378 (33.7%)	428 (36.7%)	0.88 (0.74-1.04)	0.14

* Two-sided χ^2^ test for the distributions of genotype and allele frequencies.

† Adjusted for age and body mass index.

### Stratified analysis of *FEN1* polymorphisms and BC risk

Stratified analysis regarding the effect of rs4246215 and rs174538 polymorphisms on BC by menopausal status was displayed in Table 3. The results indicated that rs4246215 was associated with a decreased BC risk in postmenopausal women (OR = 0.58, 95% CI = 0.35–0.97, *P* = 0.03). However, there was no association between rs174538 and BC risk in either premenopausal patients or postmenopausal patients. Considering the difference of BMI in cases and controls, we infer that BMI may be a important factor of BC. Then, we performed a subgroup analysis by body mass index (BMI), choosing the overweight standard 24 kg/m^2^ as a cut-point. However, we did not observe any relationship between rs4246215 and breast cancer subjects with BMI≥ 24 kg/m^2^ (*P* = 0.10) or BMI< 24 kg/m^2^ (*P* = 0.26), as shown in Table 4. Similar results were also obtained between rs174538 and BC patients.

**Table 3:** Stratified analysis by menopause status on *FEN1* polymorphisms and BC risk

rs4246215					rs174538				
Genotypes	Case (N= 560)	Control (N = 583)	*P* *	OR (95%CI)^†^	Genotypes	Case (N = 560)	Control (N = 583)	*P* *	OR (95%CI)^†^
	N (%)	N (%)				N (%)	N (%)		
**Pre-menopause**					Pre-menopause				
**G/G+G/T**	187 (89.9%)	271 (87.2%)		1.00 (reference)	G/G+G/A	297 (87.9%)	225 (84.3%)		1.00 (reference)
**TT**	21 (10.1%)	45 (12.8%)	0.16	0.68 (0.39-1.17)	AA	41 (12.1%)	42 (15.7%)	0.20	0.74 (0.47-1.18)
**Post-menopause**					Post-menopause				
**G/G+G/T**	322 (91.5%)	230 (84.7%)		1.00 (reference)	G/G+G/A	202 (90.9%)	280 (88.6%)		1.00 (reference)
**TT**	30 (8.5%)	37 (15.3%)	**0.03**	0.58 (0.35-0.97)	AA	20 (9.1%)	36 (11.4%)	0.37	0.77 (0.43-1.37)

* Two-sided χ^2^ test for the distributions of genotype frequencies.

**Table 4:** Stratified analysis by body mass index (BMI) on *FEN1* polymorphisms and BC risk

rs4246215					rs174538				
**Genotypes**	**Case N (%)**	**Control N (%)**	P^*^	**OR (95%CI)^†^**	**Genotypes**	**Case N (%)**	**Control N (%)**	P^*^	**OR (95%CI)^†^**
**BMI<24**					BMI<24				
**G/G+G/T**	479	466		1.00 (reference)	G/G+G/A	471	465		1.00 (reference)
**TT**	32	47	0.10	1.51 (0.95-2.41)	AA	41	45	0.64	1.11(0.71-1.73)
**BMI≥24**					BMI≥24				
**G/G+G/T**	30	35		1.00 (reference)	G/G+G/A	28	45		1.00 (reference)
**TT**	19	35	0.26	1.58 (0.75-3.31)	AA	20	33	0.94	1.03 (0.50-2.13)

* Two-sided χ^2^ test for the distributions of genotype frequencies.

BMI ≥24 is the judgement standard for overweight.

### Association between *FEN1* polymorphisms and clinical parameters of BC patients

To determine whether the *FEN1* polymorphisms had an effect on the different clinical features of BC patients, we then analyzed the associations between the *FEN1* polymorphisms and a series of clinic pathological parameters, including tumor size, lymph node metastasis, the status of the estrogen receptor (ER), progesterone receptor (PR), and human epidermal growth factor receptor 2 (Her-2). As shown in Table 5, we found that the mutational genotypes frequencies of the rs4246215 were significantly lower in patients with larger tumor size (> 2 cm) (heterozygote model: OR = 0.63, 95% CI = 0.44–0.92, *P* = 0.02; dominant model: OR = 0.63, 95% CI = 0.44–0.90, *P* = 0.01). However, no significant association was detected in other clinical parameters of BC patients.

**Table 5:** The associations between the FEN1 rs4246215 polymorphism and clinical characteristics of BC patients

Variables	GG (%)	TT (%)	*P* ^*^	OR (95%CI)	GT (%)	*P* ^*^	OR (95%CI)	GT+TT (%)	*P* ^*^	OR (95%CI)
**Tumor size**										
**<2 cm**	73 (38.8)	20 (10.6)		1.00 (reference)	95 (50.5)		1.00 (reference)	115 (61.2)		1.00 (reference)
**≥2 cm**	187 (50.3)	31 (8.3)	0.11	0.61 (0.32-1.13)	154 (41.4)	**0.02**	0.63 (0.44-0.92)	185 (49.7)	**0.01**	0.63 (0.44-0.90)
**LN metastasis**										
**Negative**	110 (46.6)	23 (9.7)		1.00 (reference)	103 (43.6)		1.00 (reference)	126 (53.4)		1.00 (reference)
**Positive**	150 (46.3)	28 (8.6)	0.71	0.89 (0.49-1.63)	146 (45.1)	0.83	1.04 (0.73-1.48)	174 (53.7)	0.94	1.01 (0.72-1.42)
**ER**										
**Negative**	111 (44.9)	26 (10.5)		1.00 (reference)	110 (44.5)		1.00 (reference)	136 (55.1)		1.00 (reference)
**Positive**	149 (47.6)	25 (8.0)	0.28	0.72 (0.39-1.31)	139 (44.4)	0.74	0.94 (0.66-1.34)	164 (52.4)	0.53	0.90 (0.64-1.26)
**PR**										
**Negative**	124 (48.6)	25 (9.8)		1.00 (reference)	106 (41.6)		1.00 (reference)	131 (51.4)		1.00 (reference)
**Positive**	136 (44.6)	26 (8.5)	0.86	0.95 (0.52-1.73)	143 (46.9)	0.98	1.00 (0.70-1.41)	169 (55.4)	0.34	1.18 (0.84-1.64)
**HER-2**										
**Negative**	185 (47.5)	33 (8.5)		1.00 (reference)	171 (44.0)		1.00 (reference)	204 (52.4)		1.00 (reference)
**Positive**	75 (43.9)	18 (10.5)	0.36	1.35 (0.71-2.54)	78 (45.6)	0.54	1.13 (0.77-1.64)	96 (56.1)	0.42	1.16 (0.81-1.67)

* Two-sided χ^2^ test for the distributions of genotype frequencies.

LN: Axillary lymph node; ER: Estrogen receptor; PR: Progesterone receptor; HER-2: human epidermal growth factor receptor 2.

As shown in Table 6, the same analyses were also performed for the clinical features in relation to the rs174538 polymorphism. We found that the variant genotypes of rs174538 showed a decreased association with lymph node metastasis (GA vs. GG: OR = 0.57, 95% CI = 0.39–0.81, *P* = 0.002; AA/GA vs. GG: OR = 0.61, 95% CI = 0.43–0.86, *P* = 0.005). Furthermore, the frequency of the variant genotypes of *FEN1* rs174538 polymorphism was significantly higher in ER-positive patients (GA vs. GG: OR = 1.50, 95% CI = 1.05–2.15, *P* = 0.03; AA/GA vs. GG: OR = 1.42, 95% CI = 1.01–1.98, *P* = 0.04).

**Table 6:** The associations between the FEN1 rs174538 polymorphism and clinical characteristics of BC patients

Variables	GG (%)	TT (%)	*P* ^*^	OR (95%CI)	GT (%)	*P* ^*^	OR (95%CI)	GT+TT (%)	*P* ^*^	OR (95%CI)
**Tumor size**										
**<2 cm**	72 (38.3)	20 (10.6)		1.00 (reference)	96 (51.1)		1.00 (reference)	116 (61.7)		1.00 (reference)
**≥2 cm**	171 (46.0)	41 (11.0)	0.63	0.86 (0.47-1.57)	160 (43.0)	0.22	0.79 (0.55-1.15)	201 (54.0)	0.08	0.73 (0.51-1.04)
**LN metastasis**										
**Negative**	86 (36.4)	24 (10.2)		1.00 (reference)	126(53.4)		1.00 (reference)	150 (63.6)		1.00 (reference)
**Positive**	157 (48.5)	37 (11.4)	0.57	0.84 (0.47-1.50)	130(40.1)	**0.002**	0.57 (0.39-0.81)	167 (51.5)	**0.005**	0.61 (0.43-0.86)
**ER**										
**Negative**	119 (48.2)	23 (9.3)		1.00 (reference)	105 (42.5)		1.00 (reference)	128 (51.8)		1.00 (reference)
**Positive**	114 (37.6)	38 (12.5)	0.06	1.72 (0.97-3.07)	151 (49.8)	**0.03**	1.50 (1.05-2.15)	189 (62.3)	**0.04**	1.42 (1.01-1.98)
**PR**										
**Negative**	120 (47.1)	22 (8.6)		1.00 (reference)	113 (44.3)		1.00 (reference)	135 (52.9)		1.00 (reference)
**Positive**	123 (40.3)	39 (12.8)	0.06	1.73 (0.97-3.09)	143 (46.9)	0.24	1.23 (0.87-1.76)	182 (59.7)	0.11	1.32 (0.94-1.84)
**HER-2**										
**Negative**	167 (42.9)	43 (11.1)		1.00 (reference)	179 (46.0)		1.00 (reference)	222 (57.1)		1.00 (reference)
**Positive**	76 (44.5)	18 (10.5)	0.79	0.92 (0.50-1.70)	77(45.0)	0.77	0.95 (0.65-1.38)	95 (55.5)	0.74	0.94 (0.65-1.35)

* Two-sided χ^2^ test for the distributions of genotype frequencies.

LN: Axillary lymph node; ER: Estrogen receptor; PR: Progesterone receptor; HER-2: human epidermal growth factor receptor 2.

### Haplotype analysis of *FEN1* polymorphisms and BC risk

We further conducted haplotype anaysis using the Phase 2.1 software to explore whether the interaction of rs4246215 and rs174538 SNPs affected BC risk. Compared with the G_rs4246215_G_rs174538_ haplotype, T _rs4246215_G_rs174538_ haplotype showed a decreased risk of breast cancer (OR = 0.34, 95% CI = 0.14–0.81, *P* = 0.01, shown in Table 7).

**Table 7:** The haplotype frequencies of FEN1 polymorphisms and breast cancer risk

Haplotypes		Cases (N=1120) n, %	Controls (N=1164) n, %	OR (95% CI)	*p*
rs4246215	rs174538				
G	G	735(65.6%)	718(61.7%)	1.00 (reference)	
G	A	34(3.0%)	28(2.4%)	1.19(0.71-1.98)	0.51
T	G	7(0.6%)	20(1.7%)	**0.34(0.14-0.81)**	**0.01**
T	A	344(30.7%)	400(34.4%)	0.84(0.70-1.00)	0.05

## DISCUSSION

*FEN1* is a DNA replication/repair protein with pleiotropic functions. As an important tumor suppressor, *FEN1* expression is related to the development of cancer and the progression of the disease [9, 10]. In several studies, FEN1 protein expression indicated that altered FEN1 expression might influence the therapeutic response [13]. Van Pel et al. demonstrated that *FEN1* and the flap endonuclease inhibitors have potentially broad applicability in the treatment of cancer [19].

Transient transfection and luciferase assays showed that the rs174538 G>A SNP in *FEN1* causes increased promoter activity, which is most likely to be due to a higher binding affinity of the G allele with some unknown transcriptional inhibitors. In addition, the rs4246215 G>T SNP is also associated with differential levels of *FEN1* RNA expression [16]. Interestingly, the rs174538 polymorphism is significantly associated not only with cancer risk but also with *FEN1* mRNA levels in normal tissues. Participants with the rs174538 AA genotype have been found to have significantly higher *FEN1* mRNA levels than those with the rs174538 GG and GA genotypes [20].

In our study, we observed that variant genotypes of *FEN1* rs4246215, but not rs174538, were associated with decreased BC risk. Our results are partly consistent with those of some other studies [15–18]. We searched PubMed, the ISI Web of Knowledge, Embase and China National Knowledge Infrastructure about the genome-wide association study of *FEN1*, only find one study in east China (Huaian, Jiangsu Province and Jinan, Shandong Province) about the association between the *FEN1* and breast cancer risk. The authors indicated that rs174538GG and rs4246215GG genotypes were significantly correlated to increased risk for developing breast cancer compared with the mutation homozygote [18], which were in accordance with ours. Though the samples of our study were smaller than theirs, we conducted more details subgroup analyses, which may offer evidences for clinical treatment and prognosis evaluation of breast cancer.

We also observed that the variant rs4246215 genotypes in the *FEN1* gene demonstrated a decreased association with tumor size. Furthermore, the variant genotypes of rs174538 were associated with negative lymph node metastasis and ER-positive status. These results suggested that the two polymorphisms in the *FEN1* gene are related to the development and progression of BC, and may help to accurately predict the clinical course of BC. In addition, the rs4246215 polymorphism showed an increased association with postmenopausal status. We observed that patients with cancer have lower BMI in this study, it may be inferred that patients with BC lost weight after the onset of the cancer. We conducted the stratified analysis by BMI, but we did not observe any association between BMI and the two polymorphism.

Moreover, we did haplotype anayses and find the T _rs4246215_G_rs174538_ haplotype may decrease the breast cancer risk. But, in Lv's et al. [18] study, they showed that G_rs4246215_ A_rs174538_, T_rs4246215_A_rs174538_ and G_rs4246215_G_rs174538_ haplotypes were associated with increased risks of developing breast cancer compared with the T_rs4246215_G_rs174538_ haplotype. This was broadly consistent with our study. But, we did not found G_rs4246215_A_rs174538_, and T_rs4246215_A_rs174538_ haplotypes had any associations with breast cancer. The differences may be attributed to the geographical and life style differences in northwest and east Chinese women. Thus, more large well-designed repeated studies are needed to evaluate the results.

Our study had some limitations. First, all people recruited in this study were from one hospital in the northwest China. Larger sample size and multi center experiment are needed for further verification. Second, more predisposing factors should be investigated, such as high-dose radiation exposure, and postmenopausal obesity. Third, we hypothesized that the two polymorphisms may enhance the expression of FEN1 gene via up-regulating the mRNA, thus strengthen the DNA repair ability of the injured breast cells. But, further experiments on cell or animal level are needed to explain the specific mechanisms.

In summary, our case-control study indicates that *FEN1* polymorphisms have effects in reducing the BC risk in northwest Chinese women. Further functional studies and large population-based prospective studies are still required to elucidate the influence of *FEN1* polymorphisms on BC.

## MATERIALS AND METHODS

### Ethics statement

The Institutional Review Board of the Xi'an Jiaotong University (Xi'an, China) approved the study. At the time of recruitment, written informed consent was obtained from all participants involved in the study.

### Study population

A total of 560 sporadic BC patients were recruited between January 2013 and October 2014 at the Second Affiliated Hospital of Xi'an Jiaotong University, China. The patients were recruited without age restrictions. All of the patients had pathologically confirmed. Patients were excluded if they had received chemotherapy or radiotherapy before surgery (guaranteeing the accuracy of tumor information we collected) or had other types of cancer. For comparison, 583 cancer-free controls were randomly selected from participants who were seeking health care in the outpatient departments at the hospital and were frequency-matched to the patients with age (± 5 years). All participants were interviewed with a self-administered questionnaire after obtaining the written informed consent.

### Genotyping assay

Peripheral venous blood sample of about 2 mL was collected into tubes containing ethylene diamine tetraacetie acid from each participant and then stored at −80 °C for later use. The DNA concentration was measured by spectrophotometry (DU 530 UV/VIS spectrophotometer, Beckman Coulter, Inc., Fullerton, CA, USA), as described in our previous studies [21–24]. Two tag SNPs (rs4246215 and rs174538), which captured the majority of the known common variation in *FEN1* according to the data on the Chinese population from HapMap (http://www.hapmap.org), were selected in our study. Sequenom MassARRAY Assay Design 3.0 Software (Agena Bioscience, San Diego, CA, USA) was used to design a multiplexed SNP MassEXTEND assay [25]. SNP genotyping was performed using the Sequenom MassARRAY RS1000 according to the standard protocol recommended by the manufacturer. The corresponding primers used for each SNP in our study are listed in Table 8. Sequenom Typer 3.0 Software was used for data analyses [25, 26].

**Table 8:** Primers Used for This Study

SNP_ID	1st-PCRP	2nd-PCRP	UEP_SEQ
**rs4246215**	ACGTTGGATGGATAGCA ACAAGTTTTGGAG	ACGTTGGATGCCAGC CAGGAAATCATTTCT	ATCATTTCTGTACATCTTTTGT
**rs174538**	ACGTTGGATGTTTGCCCCGCG AAGGCTAAT	ACGTTGGATGACTTC CTTTTCCGGTTGTGC	ttCTAGGCGCCTGCTCCTG

### Statistical analyses

The Student t-test or the χ^2^ test was used to examine the differences in the distributions of demographic characteristics, selected variables, and genotypes distributions of the two SNPs between the patients and controls. Hardy-Weinberg equilibrium (HWE) was tested by chi-square test for each SNP before the analysis. We conducted a case-control study for all of the subjects with adjustment for age and body mass index (BMI), and then performed the stratified analyses by menopausal status and BMI. The associations between *FEN1* rs4246215 and rs174538 polymorphisms and breast cancer susceptibility were estimated by odds ratios (ORs) and 95% confidence intervals (CIs). Corresponding primers used for each SNP in our study are listed in Table 8. Five genetic models were used in our study, namely allele model, co-dominant model (contains of homozygote model and heterozygote model), recessive model, dominant model, and over-dominant model. All statistical analyses were performed with SPSS 18.0 for Windows software (PASW Statistics, SPSS Inc., Chicago, IL, USA). A *P*-value < 0.05 was considered as the criterion of statistical significance, and all statistical tests were two-sided. Phase2.1 software (download from http://stephenslab.uchicago.edu/phase/download.html) was used to conduct all common haplotypes and χ^2^ test was used to estimate the ORs and 95% CIs for each haplotype.
